# Identification of Dinaciclib and Ganetespib as anti-inflammatory drugs using a novel HTP screening assay that targets IFNγ-dependent PD-L1

**DOI:** 10.3389/fimmu.2025.1502094

**Published:** 2025-04-08

**Authors:** Shira Hagbi-Levi, Michal Abraham, Lika Gamaev, Inbal Mishaelian, Ophir Hay, Elina Zorde-Khevalevsky, Ori Wald, Hanna Wald, Devorah Olam, Lola Weiss, Amnon Peled

**Affiliations:** ^1^ Goldyne Savad Institute of Gene Therapy, Hadassah-Hebrew University Medical Center, Jerusalem, Israel; ^2^ AlonBio Ltd., Ness Ziona, Israel

**Keywords:** IFNγ, PD-L1, CXCL10, immunotherapy, cancer, autoimmunity, inflammation, drug discovery

## Abstract

**Introduction:**

IFNγ plays both positive and negative roles in the regulation of innate and adaptive immune responses against tumors and virally infected tissues by upregulating CXCL10 and PD-L1 expression.

**Methods:**

To identify novel pathways and drugs that regulate the IFNγ-dependent PD-L1, we expressed GFP under the control of mouse PD-L1 promoter in mouse cancer cells that up regulate PD-L1 and CXCL10 in response to IFNγ stimulation. Using these cells, we screened an FDA approved library of 1496 small molecules known for their ability to inhibit IFNγ-dependent increase in PD-L1.

**Results:**

We identified 46 drugs that up regulated and 4 that down regulated IFNγ-dependent PD-L1 expression. We discovered that in addition to the known JAK inhibitors Ruxolitinib and Baricitinib, Dinaciclib, a CDK1/2/5/9 inhibitor, and Ganetespib, a Hsp90 inhibitor, significantly inhibit both PD-L1 and CXCL10 expression in the model cells. Furthermore, both drugs suppressed IFNγ-dependent CXCL10 and PD-L1 expression *in-vitro* in primary human lung cells and human cancer cells. These drugs also significantly inhibited delayed-type hypersensitivity (DTH) *in-vivo* in an inflammation mouse model.

**Discussion:**

Our novel screening platform can therefore be used in the future to identify novel immunomodulators and pathways in cancer and inflammation, expanding therapeutic horizons.

## Introduction

Immunotherapy becomes necessary when the immune system exhibits abnormal responses, either targeting healthy cells, as in autoimmune diseases or viral infections, or failing to recognize and attack abnormal cells, such as cancerous ones. In such scenarios of immune dysfunction, Interferon-gamma (IFNγ), a crucial immuno-regulatory cytokine, plays a pivotal role with dual biological functions.

IFNγ is a pleiotropic cytokine produced mainly by natural killer (NK) cells, and activated T cells including NKT cells, and monocytes that plays a central role in promoting innate and adaptive mechanisms of host defense by immune regulation (1, 2). The biological actions of IFNγ are particularly broad because almost all normal cells express functionally active IFNγ receptors on their surfaces (3).

IFNγ activates the innate and specific immunity against virus-infected cells (4, 5). In addition to being a crucial regulator of overall inflammatory responses to pathogens, IFNγ is a well-known broad-spectrum anti-microbial agent (6). Many antiviral proteins, induced by IFNγ, help in countering numerous viral infections at several stages, such as in viral entry, un-coating, blocking viral translation or virion assembly. Moreover, IFNγ deficiency in mice may cause a high susceptibility to infections due to intracellular bacteria in the context of virally infected tissues (7).

Activation of the immune system and the consequent production of inflammatory cytokines are essential for the innate anti-viral immune responses. However, hyper-activation of the immune system results in an acute increase in circulating levels of pro-inflammatory cytokines, leading to a “cytokine storm” (8). A relevant example of a condition characterized by a “cytokine storm” is COVID-19. Acute respiratory distress syndrome (ARDS) and systemic inflammatory response syndrome (SIRS) are other serious consequences of cytokine storm (9).

The dual and opposing role of IFNγ is reflected not only in viral infection but in cancer development as well. IFNγ is a key cytokine in the polarization and recruitment of Th1 (CD8+ T cells). It upregulates the chemokines CXCL9 and CXCL10 that attract the cytotoxic T-cells, NK and NKT cell into tumors (10, 11). Furthermore, IFNγ deficiency in mice may cause development of lymphoma or lung epithelial malignancies (12).

In parallel to promoting innate and adaptive mechanisms of host defense, IFNγ is highly involved in tumor control (13). It negatively regulates the magnitude of immune response by upregulating the immune checkpoint cell surface receptor programmed death-ligand 1 (PD-L1), a phenomenon called “adaptive resistance” (14, 15). Upregulation of PD‐L1 expression is a strategy exploited by tumor cells to escape antitumor immunity. However, by upregulating PD-L1 and CXCL10, IFNγ directly enhances and reduces the immunogenicity of tumor cells (16–18). It was found that patients with tumors, all expressing IFN‐g, CXCL10 and PD‐L1 have the best prognosis for anti- PD-1/PD-L1 treatments compared to those with tumors not expressing IFN‐g, CXCL10 or PD‐L1 (19, 20). Moreover, resistance to immunotherapy is attributed to defects in IFNγ signaling (21).

In addition to viral infections and malignancies, IFNγ is also involved in autoimmune diseases because of its ability to disrupt the immune system homeostasis. For example, insulin-producing β cells respond to pancreatic inflammation and IFNγ production by upregulating PD-L1 expression to limit self-reactive T cells (22). In addition, genome wide-association studies (GWAS) identified IFNγ and IFNγ-inducible genes as loci that contribute to the susceptibility to connective tissues diseases (CTDs), such as lupus (23, 24). Furthermore, the most prevalent side effect for anti- PD-1/PD-L1 therapies is the breakout of autoimmunity in treated patients (25).

This study proposes a novel screening platform for identifying small molecules targeting IFNγ-dependent PD-L1 and CXCL10 expression. Such a screening platform holds promise for identifying novel pathways and drugs that target inflammation and cancer.

## Materials and methods

### Cell lines and plasmids

The majority of cell lines (RENCA, B16F10, 3LL, AB12, PANC1) were acquired from the American Type Culture Collection (ATCC; Manassas, VA), with the exception of the LivMet cell line, which was generously provided by Prof. David Tuveson. LivMet cells are derived from mouse liver metastases that arose in KrasG12D/+ transgenic mice, which developed pancreatic ductal adenocarcinoma (PDA) tumors.

The LPA stable cell line was established in our laboratory. We engineered a green fluorescent protein (eGFP) plasmid vector (9600 bp) containing the mouse PD-L1 (CD274) promoter (1629 bp: 1341 bp downstream and 287 bp upstream of the transcription splice site), the eGFP gene, and puromycin and ampicillin resistance genes (**Supplementary Figure 1**). This plasmid was custom-designed and synthesized by GenCopeia™ (Rockville, MD) according to our specifications.

GeneCopeia utilized the GeneCopeia Lenti-Pac™ HIV Expression Packaging Kit to co-transfect a GeneCopeia Lenti-Pac HIV Expression Packaging plasmid with the HIV-based lentiviral expression plasmid into GeneCopeia 293T lentiviral packaging cells. This process yielded pseudovirus particles containing the lentiviral expression construct. GeneCopeia provided a titer of 2.82*10^8 TU/ml lentivirus along with the necessary plasmid. Lentiviruses were shipped on dry ice and stored at -80°C until use.

### Viral infection

To establish a stable cell line expressing the PD-L1-probe, we integrated the viral expression construct into the genomic DNA of LivMet cells. Initially, 3*10^4 cells were seeded in a 48-well plate and reconstituted with DMEM (Sartorius) containing 10% FCS (Gibco), 1% L-glutamine (Sartorius), 1% penicillin-streptomycin (Gibco), and 1% sodium pyruvate (Gibco). 24 hours later, the medium was replaced with the same medium containing 1% FCS, and cells were infected with 1.5µl of lentivirus (MOI=10). Four hours post-infection, 10% FCS medium was added, and cells were incubated for four days before selection with 4 µl/ml puromycin (Tivan Biotech). Following six additional days of incubation, surviving cells were seeded into ten 96-well plates at a concentration of 0.5cell/well.

**Figure 1:**
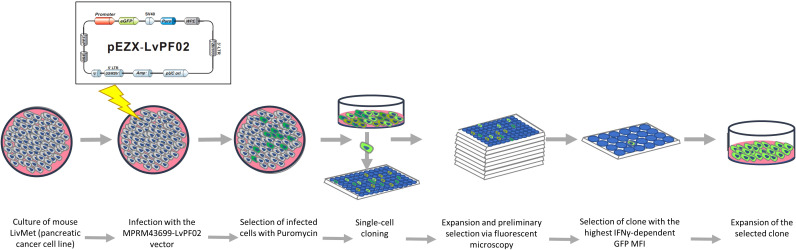
A schematic setup of lentivirus infection and the production of the LPA cell line.

Following a week of incubation, using a fluorescent microscopy, all wells were screened, and those containing only one colony were selected for further assessment of eGFP expression. Clones demonstrating high eGFP expression prior to IFNγ stimulation, potentially indicating plasmid insertion in an overexpressed genomic region, were excluded from further examination. Conversely, clones showing minimal or no eGFP expression were split into two groups and subjected to IFNγ stimulation. After stimulation with 20ng/ml mouse IFNγ (PeproTech) for 48 hours, eGFP mean fluorescent intensity (MFI) was measured via flow cytometry. The clone exhibiting the most significant response to IFNγ 48hours post-stimulation (LPA), was identified and chosen for the high throughput screening assay (**Figure 1**).

### Screening procedure

A library comprising 1496 FDA-approved drugs was sourced from ApexBio (Houston, TX, USA). Each compound was initially dissolved in 100% DMSO at a concentration of 10 mM. For screening, each compound was used at a working concentration of 10 μM, with and without the addition of 20ng/ml mouse IFNγ. Testing was performed at 24-hour and 48-hour time points post-stimulation, utilizing four separate plates: no IFNγ for 24hours, no IFNγ for 48 hours, IFNγ for 24hours and IFNγ for 48 hours.

Compounds and/or IFNγ were dissolved in the cell medium and added to the culture 24 hours after seeding at 1*10^4 LPA cells per well. The final concentration of DMSO in the culture was 0.1%. Each plate consisted of 8 control wells: 2x LivMet, 2x LivMet+IFNγ, 2x LPA, and 2x LPA+IFNγ. Cells were harvested using 0.25% trypsin-EDTA (Sartorius), which was then neutralized with DMEM supplemented with 10% FCS. Following this, cells underwent three washes with 1xPBS to remove any remaining supernatant.

**Figure 2:**
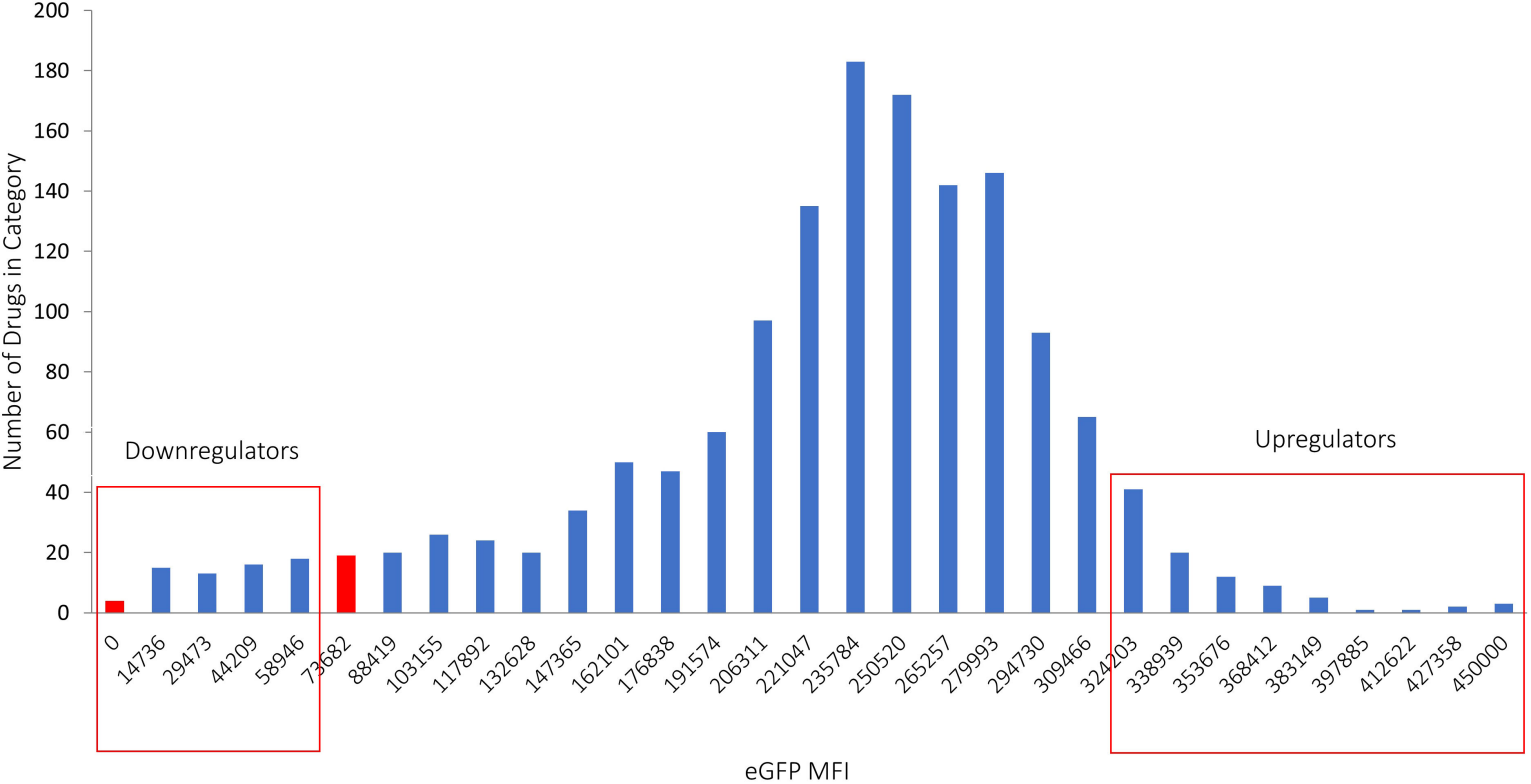
Hits identified in the screening process. Drugs that caused a decrease in eGFP MFI below the level observed in IFNγ-treated cells without drugs were classified as down-regulators, while drugs that led to an increase in eGFP MFI exceeding 2 standard deviations above the average were categorized as up-regulators. The X axis represents the classification of different MFI levels resulting from IFNγ treatment. The Y axis shows the number of drugs in each category, illustrating the distribution of drug effects on eGFP expression.

Finally, cells were resuspended in 1xPBS, and the eGFP MFI was measured using flow cytometry. Compounds that resulted in eGFP MFI values greater than 2 standard deviations compared to the averaged MFI in the specific plate were identified as “hits” that up-regulate PD-L1. Conversely, compounds that decreased eGFP MFI to a level lower than that of control IFNγ-treated LPA cells without any drug were considered “hits” that down-regulate PD-L1. This scoring approach was chosen due to the significantly higher averaged eGFP MFI of a plate compared to the IFNγ-treated control with no drug. Drugs were categorized according to their effect on the expression of IFNγ-dependent eGFP expression (under the control of the PD-L1 promotor) (**Figure 2**).

All hits were subjected to further validation on LivMet cells across three concentrations to assess dose responses (0.1, 1, and 10μM). Initially, 1*10^4 LivMet cells were seeded per well in a 96-well plate, and after 24hours, IFNγ with either 0.1, 1, or 10μM of each drug were added. Cell supernatants were collected 48 hours following treatment and stored at -20°C until further use. Additionally, cells were stained with an anti-mouse CD274 (PD-L1, B7-H1) antibody (eBioscience; CA, USA) for subsequent flow cytometry analysis. The supernatant was also tested for mouse CXCL10 secreted levels using a DuoSet enzyme-linked immunosorbent assay (ELISA) kit (R&D Systems; MN, USA).

### Human primary lung cells

A human primary lung tissue was collected from 5 healthy lung donors. All donors signed an informed consent form, and the study was approved by the institutional ethics committee (see Ethics Declaration Helsinki number- HMO-21-235). Lung tissues were washed with ice-cold 1xPBS plus 1.6% penicillin/streptomycin. Tissue was sliced into 1 mm slices and centrifuged in 1500 rpm for 5 min in 4°C. Supernatant was removed, and cells were re-suspended in a 10 ml cell dissociation buffer comprising: 80% RPMI (Sartorius); 0.4% BSA (Sigma-Aldrich); 0.1% collagenase P (Sigma-Aldrich) and 0.01% DNase (Sigma-Aldrich). Cells were incubated in a 37°C water bath for 30 min and pipetted every 10 min until full cell dissociation of the tissue. Then, we filtered the suspension through a 70-micron filter and enzymes were deactivated with RPMI+10% FCS. After filtration, cells were centrifuged and washed with 10ml RPMI. Following an additional centrifugation round, cells were re-suspended in 500 μl of 1xACK (Gibco) for 2.5min and washed twice with 10ml RPMI. Primary lung cells were seeded in a collagen-coated tissue culture for 48 hours until adherence to plate. Then, medium was replaced with a fresh RPMI medium containing 10% FCS, 1% L-glutamine, 1% penicillin -streptomycin and 1% sodium pyruvate. Cells were collected with 0.25% trypsin-EDTA and 1*10^4 cells per well were seeded in a 96-well plate. 24 hours later, cells were treated with IFNγ and the candidate drugs. After 72hours of incubation, supernatant was collected for assessing CXCL10 and IL-6 levels with ELISA, and cells were washed with 1xPBS and split into two groups. One group was stained with anti-human Epcam (Biolegend), anti-human MHC1 (Biolegend) and anti-human PD-L1 (Biolegend), while the other group was stained with anti-human CD45 (Biolegend), anti-human MHC1 and anti-human PD-L1.

### Human cancer cells

Human pancreatic cancer cell line Panc1 was used to test the effect of 11 selected drugs *in-vitro* in a human cell line. Panc1 cells were cultured in RPMI medium containing 10% FCS, 1% L-glutamine, 1% penicillin-streptomycin and 1% sodium pyruvate. 2*10^4 cells were seeded per well in a 96-well plate, and after 24hours, 20ng/ml human IFNγ with 1 or 10μM of each drug, in triplicates, were added. Cell supernatants were collected 48 hours following post-treatment and stored at -20°C until further use. Additionally, cells were stained with an anti-human CD274 (PD-L1, B7-H1) antibody (BioLegend) for subsequent flow cytometry analysis. The supernatant was also tested for human CXCL10 secreted levels using a DuoSet enzyme-linked immunosorbent assay (ELISA) kit (R&D Systems; MN, USA).

### Delayed-type hypersensitivity mouse model

For the DTH model we used female BALB/cOlaHsd mice (Envigo). Mice were sensitized over their shaved abdominal skin with 100µl of 2% 4-Ethoxymethylene-2-phenyl-2-oxazalin-5-one (Sigma-Aldrich) dissolved in acetone/olive oil [4:1 (vol/vol)] applied topically (day 0). DTH sensitivity was elicited 6 days later by challenging the mice with 20µl of 0.5% oxazalone reconstituted in acetone/olive oil, with 10µl being administered topically to each side of their right ears. Ear thickness was measured 24 hours after the challenge using a micrometer digital caliper (Mitutoyo Corp; Tokyo, Japan). Thickness of the left ear served as the control for each mouse (26). Positive control mice were subcutaneously injected with dexamethasone (Omega) (100ug/mouse in a total volume of 200µl) on day 5 following challenge (n=5-8 mice per experiment). Negative control mice were treated daily subcutaneously with 1xPBS. Clofazimine (Sigma-Aldrich) was administered by a gavage needle per OS (300mg/kg) on days 2,4,5 and 6 following challenge (n=7). Dinaciclib (Abcam) was injected intraperitonially (400ug/mouse/inj) on days 0, 3 and 6 following challenge (n=6 and 7 in two different experiments). Penfluridol (Sigma-Aldrich) was administered by a gavage needle per OS (10mg/kg/inj) on days 3,4,5 and 6 following challenge (n=5). Nefiracetam (Abcam) was injected intraperitonially (1mg/kg/inj) on days 3, 4, 5 and 6 following challenges (n=6). Baricitinib (Selleckchem) was administered by a gavage needle per OS (10mg/kg/inj) on days 3,4,5 and 6 following challenge (n=7). Ganetespib (Abcam) was administered IV (500ug/mouse) on day 6 following challenge (n=5). Cyclosporin A (Tocris) was administered intraperitonially (100ug/mouse/inj) on days 0, 3 and 6 following challenge (n=7). Glycopyrrolate (Sigma-Aldrich) was injected subcutaneously (1.5mg/kg) on days 3 and 6 following challenge (n=7). Deferasirox (Selleckchem) was administered by a gavage needle per OS (30mg/kg/inj) on days 3,4,5 and 6 following challenge (n=5). Pizotifen (Adooq Bioscience) was injected intraperitonially (10mg/kg/inj) on days 4, 5 and 6 following challenge (n=8). Axitinib (Sigma-Aldrich) was administered by a gavage needle per OS (10mg/kg/inj) on days 3,4,5 and 6 following challenge (n=7). Cinepazide maleate (Sigma-Aldrich) was administered intraperitonially (20mg/kg/inj) on days 3, 4, 5 and 6 following challenge (n=7). Experiments were conducted with the approval of the institutional animal care ethics committee (See Ethics Declaration). The percent change in ear thickness of each mouse was calculated. Subsequently, the fold change of each mouse compared to the averaged percent change of the control group in each experiment was determined. The mean fold change ± standard error of the mean was then calculated. A p-value < 0.05 was considered statistically significant.

### Flow cytometry

Anti-mouse CD274 (PD-L1, B7-H1) antibody PE-conjugated clone MIH-5 (eBioscience™) was used at a 1:50 dilution and was added to the cells following a 1xPBS wash. Cells were incubated for 30min with the antibody and washed and data were collected on a CytoFLEX instrument (Beckman Coulter). MFI of PE was analyzed using CytExpert 2.4 software. The background was defined as the MFI of the isotype control rat IgG2a kappa Isotype Control (eBR2a), PE (eBioscience; CA, USA). eGFP MFI of LPA cells was measured by the laser 488 filter 525-40 of the CytoFLEX instrument and was analyzed using CytExpert 2.4 as well.

### Quantitative polymerase chain reaction

RNA was extracted from cells using an RNA-isolation reagent (TriReagent; Sigma-Aldrich) according to the manufacturer’s protocol. RNA was then treated with DNase (TURBO DNA-free, Ambion). mRNA quantity was assessed using Nanodrop (Thermo Scientific). Reverse transcription of RNA to cDNA was performed using the qScript™ cDNA synthesis kit (Quanta Biosciences; Gaithersburg, MD). qPCR was performed with PerfeCta^®^ SYBR^®^ Green FastMix^®^ ROX on a BioRAD CFX384™ Real-Time System according to the manufacturer’s protocol. Cycling conditions were 95°C for 20s, followed by 40 cycles of 95°C for 1s, and 60°C for 20s, 65°C for 5s. mRNA expression of PD-L1 (sense- TAATCAGCTACGGTGGTGCG; anti-sense- CTTCTCTTCCCACTCACGGG), CXCL10 (sense- GAGAGACATCCCGAGCCAAC; anti-sense- GGGATCCCTTGAGTCCCAC) and eGFP (sense- GGTCACGAACTCCAGCAG; anti-sense- CAGAAGAACGGCATCAAGG) were evaluated in triplicates and were normalized to the expression levels of the endogenous control HPRT1 (sense- AGGGCATATCCAACAACAAACTT; anti-sense- GTTAAGCAGTACAGCCCCAAA) and calculated according to the standard formula of 2^(-ΔΔCT)^, producing results as a relative quantification (RQ).

### Statistical analyses

Data are presented as the mean ± SD. Statistical comparisons of the means were performed using two-tailed unpaired Student’s *t*-tests. Differences of *p* ≤ 0.05 were considered statistically significant.

## Results

### The effect of IFNγ on PD-L1 and CXCL10 is cell-type dependent

While IFNγ activates the immune response by stimulating CXCL10 expression, it also increases the levels of the immune checkpoint ligand PD-L1 which suppresses the immune response. This dual effect negatively regulates the immune response. To investigate this phenomenon, we sought for cell types that upregulate the expression of PD-L1 and CXCL10 in response to IFNγ stimuli.

**Figure 3:**
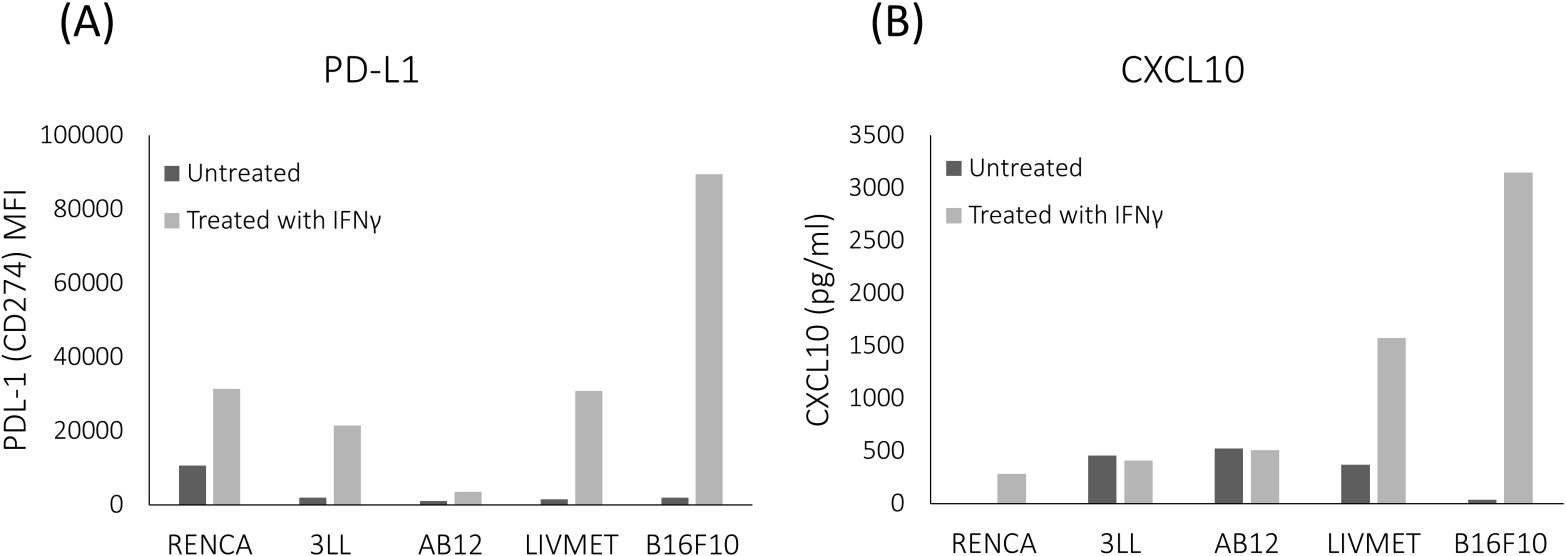
Comparative analysis of the impact of IFNγ stimulation across various murine cell lines. LivMet and B16F10 cells exhibited a more robust response to IFNγ stimulation compared to RENCA, 3LL, and AB12 cells. **(A)** Flow cytometry analysis showing PD-L1 protein levels (measured as MFI of PE) in the five tested cell lines after IFNγ treatment versus untreated controls. **(B)** ELISA results showing CXCL10 protein levels (measured in pg/ml) in the same cell lines following IFNγ treatment compared to untreated controls.

Screening of various murine cell lines revealed differences in the expression of PD-L1 and CXCL10 following activation by IFNγ. Among the cell lines tested were RENCA (carcinoma), 3LL (Lewis lung carcinoma), AB12 (mesothelioma), B16F10 (melanoma), and LivMet (a liver metastasis of pancreatic cancer cell line derived from KrasG12D/+ transgenic mice with pancreatic ductal adenocarcinoma (PDA) tumors). Notably, LivMet cells exhibited a substantial upregulation of both CXCL10 and PD-L1 upon IFNγ stimulation (4.26-fold and 20.83-fold, respectively), compared to untreated controls. In contrast, 3LL, RENCA, and AB12 cells showed lower levels of CXCL10 post-stimulation (407pg/ml, 281pg/ml and 509pg/ml, respectively) compared to control untreated cells (456pg/ml, 0pg/ml and 523pg/ml, respectively), while LivMet and B16F10 cells displayed elevated CXCL10 levels (1572pg/ml and 3145pg/ml, respectively) compared to control untreated cells (366pg/ml and 37pg/ml, respectively) (**Figure 3**). Increased PD-L1 expression following IFNγ stimulation was evident in all tested cell lines.

Given the strong response of LivMet cells to IFNγ, with significant elevations in both PD-L1 and CXCL10 levels, we selected this cell line for our high-throughput screening (HTS) assay. This robust response makes LivMet cells particularly suitable for evaluating the effects of various drugs on IFNγ-dependent PD-L1 and CXCL10 expression. Our goal was to assess whether these cells could reliably exhibit both upregulation and downregulation of these markers in response to different compounds.

**Figure 4:**
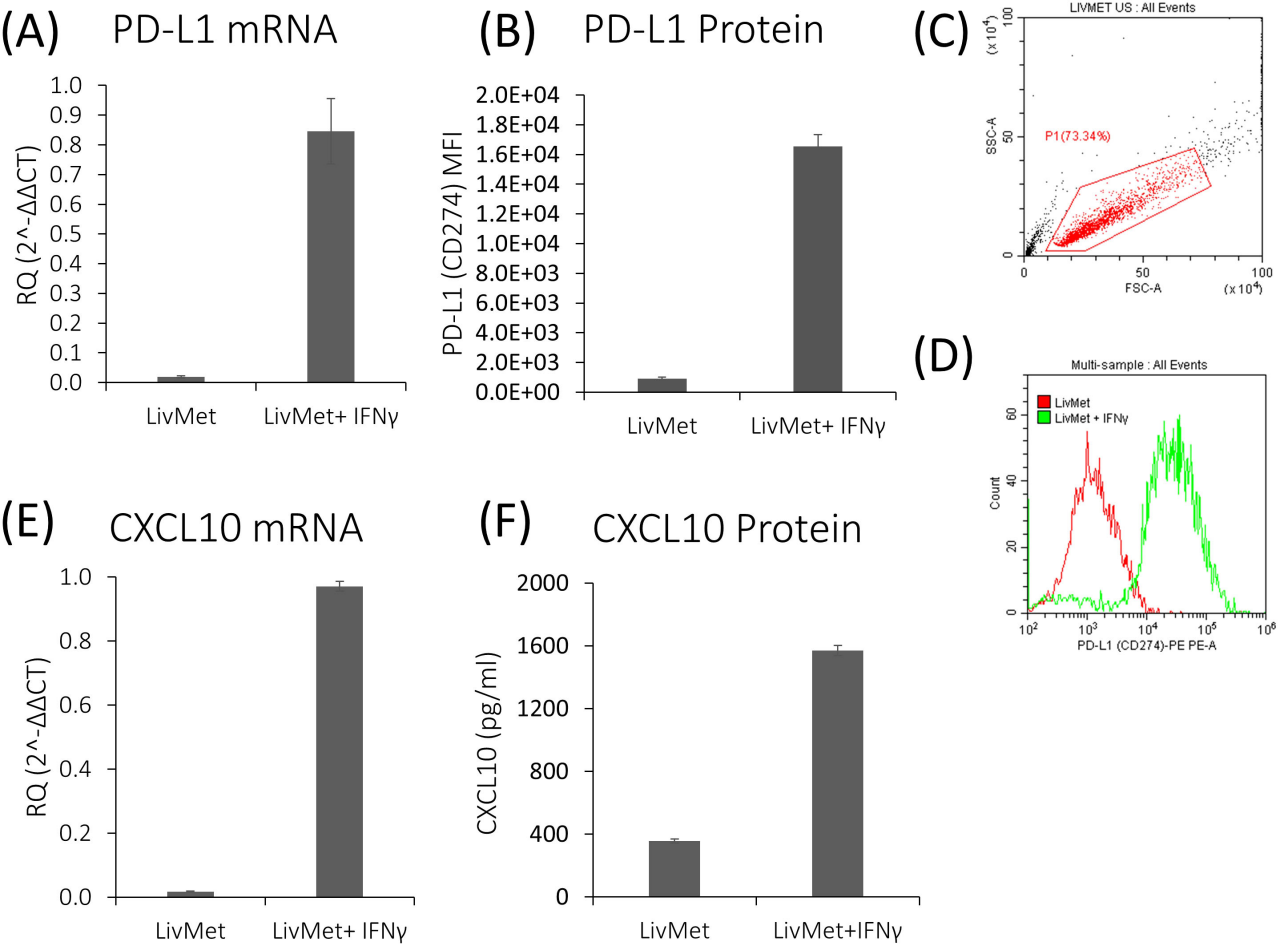
IFNγ-dependent genes are upregulated following stimulation of LivMet cells with IFNγ. **(A)** qPCR analysis of PD-L1 mRNA expression (RQ, Relative Quantification) in LivMet cells treated with IFNγ compared to untreated control cells. **(B–D)** Flow cytometry analysis of PD-L1 protein levels measured as MFI of PE, in LivMet cells at 48 hours post-IFNγ treatment compared to untreated controls. **(E)** qPCR analysis of CXCL10 mRNA expression (RQ) in IFNγ-treated LivMet cells compared to untreated controls. **(F)** ELISA analysis of CXCL10 protein levels (pg/ml) in the supernatant of IFNγ-treated LivMet cells compared to untreated controls.

Using a combination of flow cytometry, qPCR, and ELISA measurements, we demonstrated that LivMet cells uphold the necessary molecular machinery for IFNγ-dependent PD-L1 and CXCL10 expression. mRNA and protein levels of PD-L1 were highly expressed 48 hours after IFNγ stimulation (44.7-fold, P val=0.006 and 18.22-fold, P val<0.0001; respectively), in addition to CXCL10 mRNA and protein levels which were elevated as well (54.65-fold, P val<0.0001 and 4.4-fold, P val<0.0001; respectively) (**Figure 4**). This indicates their potential as a tool for assessing the impact of drugs on IFNγ-dependent immunity.

### Molecular sensor for IFNγ-dependent PD-L1 expression

To facilitate the screening of a vast library of small molecules, we engineered an endogenous molecular sensor integrated into LivMet cells. This sensor, constructed within a lentiviral vector, pEZX-LvPF02 (GeneCopeia), features a reporter gene, i.e., eGFP, controlled by the murine PD-L1 (CD274) promoter and incorporating puromycin resistance (see **Supplementary Figure 1**). The expression levels of eGFP serve as an indication for PD-L1 activation. LivMet cells were transduced with the lentiviral vector, followed by single-cell cloning to isolate the most responsive clone exhibiting IFNγ-dependent eGFP expression (See Materials and Methods). The novel LivMet cell line is termed “LPA”.

### HTS approach reveals candidate drugs modulating PD-L1 expression

**Figure 5:**
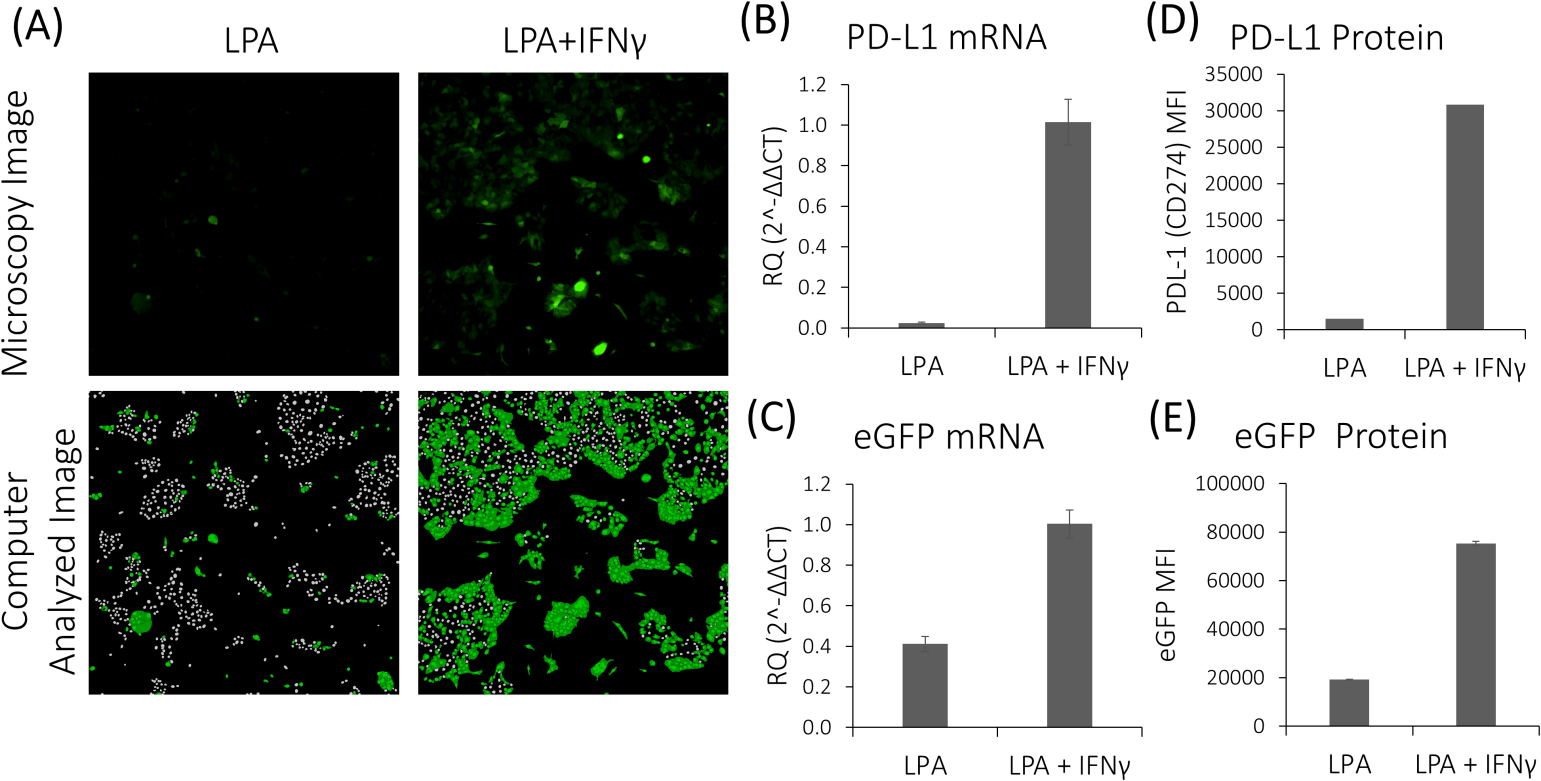
Response of LPA cells to stimulation with IFNγ. **(A)** Fluorescence microscopy and computer image analysis of untreated and IFNγ-treated LPA cells. **(B,C)** qPCR analysis reveals increased PD-L1 and eGFP mRNA expression levels in LPA cells following IFNγ stimulation. **(D, E)** ELISA analysis demonstrates elevated PD-L1 and eGFP protein levels in LPA cells following IFNγ stimulation.

To assess the efficacy of different compounds in modulating IFNγ-dependent PD-L1 activation, we utilized HTS approach on the newly established LPA cell line. Compounds altering PD-L1 expression were also evaluated for their impact on CXCL10 secretion. First, we validated IFNγ-dependent PD-L1 activation in LPA cells by examining eGFP expression. Fluorescence microscopy analysis revealed approximately a 40% increase in eGFP expression in LPA cells post-IFNγ stimulation (**Figure 5A**). Moreover, flow cytometry and qPCR analyses demonstrated that the expression levels of both PD-L1 and eGFP in LPA cells were elevated following IFNγ stimulation, with mRNA expression levels increasing by 42.6-fold and 2.45-fold, and protein levels by 20.83-fold and 3.9-fold, respectively (**Figures 5B–E**).

After confirming the suitability of LPA cells for identifying small molecules targeting IFNγ-mediated PD-L1 expression, we screened 1496 drugs from the DiscoveryProbe™ FDA-approved drug library (ApexBio) to evaluate their effect on IFNγ-dependent PD-L1 expression. Initially, all candidate drugs were screened at a concentration of 10 μM, and the eGFP MFI was evaluated 48 hours post-IFNγ stimulation. Additionally, drugs were assessed for their effect on eGFP MFI without IFNγ stimulation. This analysis revealed 130 hits, of which 48 drugs downregulated IFNγ-dependent eGFP expression, and 82 drugs upregulated it.

### Validation of hits modulating PD-L1 and CXCL10 protein expression in a mouse LivMet cell line

**Figure 6:**
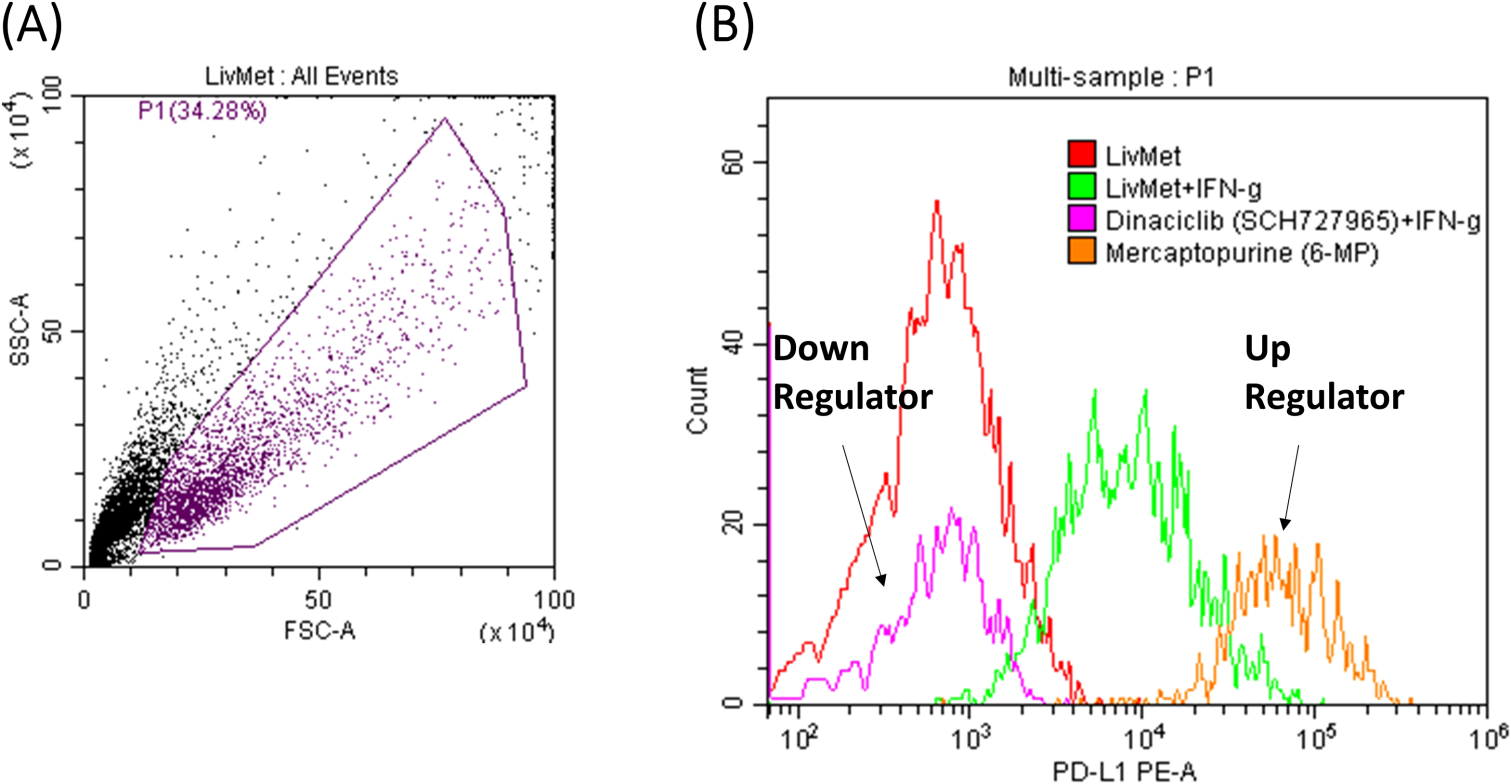
FACS results of two representative drugs from the validation set. **(A)** Foreword-side scatter of LivMet cells. **(B)** Histogram comparing the MFI levels of anti- PD-L1 PE. The histogram depicts untreated LivMet cells (Red), LivMet cells treated with IFNγ (Green); LivMet cells treated with IFNγ and Dinaciclib which downregulates IFNγ-dependent PD-L1 activation (Magenta); and LivMet cells treated with IFNγ and Mercaptopurine, which upregulates IFNγ-dependent PD-L1 activation (Orange).

Following the identification of 130 potential drug candidates, we proceeded with validating their effect, down- or up-regulation of PD-L1 expression. This was achieved by performing a drug dose response on unmodified LivMet cells, in increasing drug concentrations of 0.1, 1 and 10μM, and PD-L1 staining, assessed via flow cytometry (**Figure 6**). Of the 130 hits, 104 drugs were validated to alter PD-L1 protein levels in LivMet cells, demonstrating an 80% validity of the screening assay. Subsequently, 10 drugs among the validated 104 were found to be cytotoxic for LivMet cells (with less than or equal to 10% of live cells at 1μM concentration), and thus, were excluded from further analysis.

The quality of the screening was assessed by two factors: The Z’ factor and the signal-to-noise ratio. The Z’ factor, calculated as Z’ = 1 − 3 SD of positive control + 3 SD of negative control/|mean of positive control − mean of negative control|, was determined to be 0.694, indicating an excellent assay quality. Similarly, the signal-to-noise ratio (S/N = (mean signal – mean background)/SD of background) was calculated to be 71.2, further confirming the robustness of the assay and the effective separation of the distributions in the screening method.

We excluded drugs with minor effects on PD-L1 protein levels (Log2FC between -0.2 and 0.2), resulting in a selection of 50 drugs. Subsequently, these 50 drugs were further evaluated for their impact on CXCL10 expression in the supernatant of treated LivMet cells. The 50 hits were further classified into four groups based on their potential therapeutic utility (**Table 1**):

**Table 1:** List of the 50 validated and non-toxic drugs that demonstrated an effect on IFNγ-dependent PD-L1 and CXCL10 expression.

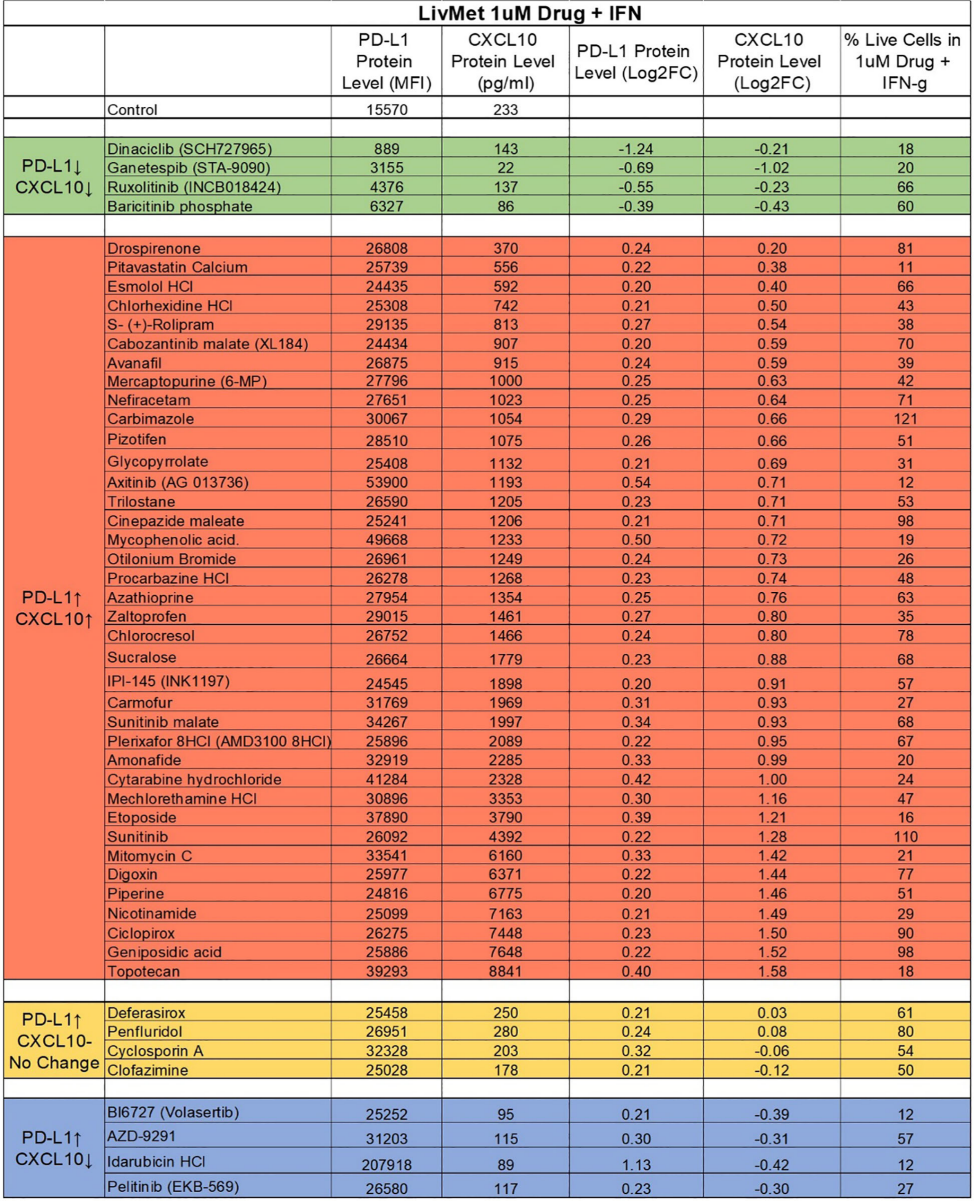

These drugs were categorized into four groups based on their exuded effect: Group 1 (Green), downregulation of both PD-L1 and CXCL10; Group 2 (Red), upregulation of both PD-L1 and CXCL10; Group 3 (Yellow), upregulation of PD-L1 with no effect on CXCL10 expression; and Group 4 (Blue), upregulation of PD-L1 with downregulation of CXCL10. Changes in protein levels were considered significant when Log2FC was ≤ -0.2 (indicating downregulation) or ≥ 0.2 (indicating upregulation). Compounds that had no effect on IFNγ dependent PD-L1 expression and cytotoxic compounds (cells%≤10%) were excluded.

Group 1: Comprises four drugs that downregulate both PD-L1 and CXCL10. These drugs hold promise for treating conditions such as viral infections, graft-versus-host disease (GVHD), inflammation, and autoimmunity, where promoting a less immunogenic environment is desirable.

Group 2: Consists of 38 drugs that upregulate both PD-L1 and CXCL10 expression. Group 3: Four drugs that upregulate PD-L1 without affecting CXCL10.

Groups 2 and 3 present potential candidates for treating immune-related diseases or disorders like hyperinflammatory syndrome in viral infections (such as in COVID-19), GVHD, and cancer, where the recruitment of cytotoxic T cells is pivotal, and PD-L1 overexpression enhances immunogenicity, warranting the use of anti- PD-L1 therapeutics in combination with identified drugs.

Group 4: Comprises four drugs that upregulate PD-L1 but downregulate CXCL10. These drugs are potential candidates for treating immune-related diseases or disorders such as autoimmunity, inflammation, and hyperinflammatory syndrome in viral infections (such as in COVID-19), where the aim is to suppress the recruitment and activation of cytotoxic T cells.

### Effect of Dinaciclib and Ganetespib on IFNγ-dependent PD-L1 and CXCL10 expression in human normal primary lung cells and cancer cells

After their validation in mouse tumor cells (LivMet), we focused next on human cells. We assessed all four drugs of Group 1 (Ruxolitinib, Baricitinib, Dinaciclib and Ganetespib) that were shown to downregulate IFNγ-dependent PD-L1 and CXCL10 expression in mice cells, in addition to 7 representatively selected drugs from the second and third groups, in human normal primary lung cells. Representative drugs that upregulate IFNγ-dependent PD-L1 expression and either upregulate (Group 2: Glycopyrrolate, Axitinib, Nefiracetam, Cinepazide maleate) or have no effect on (Group3: Deferasirox, Cyclosporin A, Clofazimin) IFNγ-dependent CXCL10 expression. Group 4 was out of our focus in this study.

**Figure 7:**
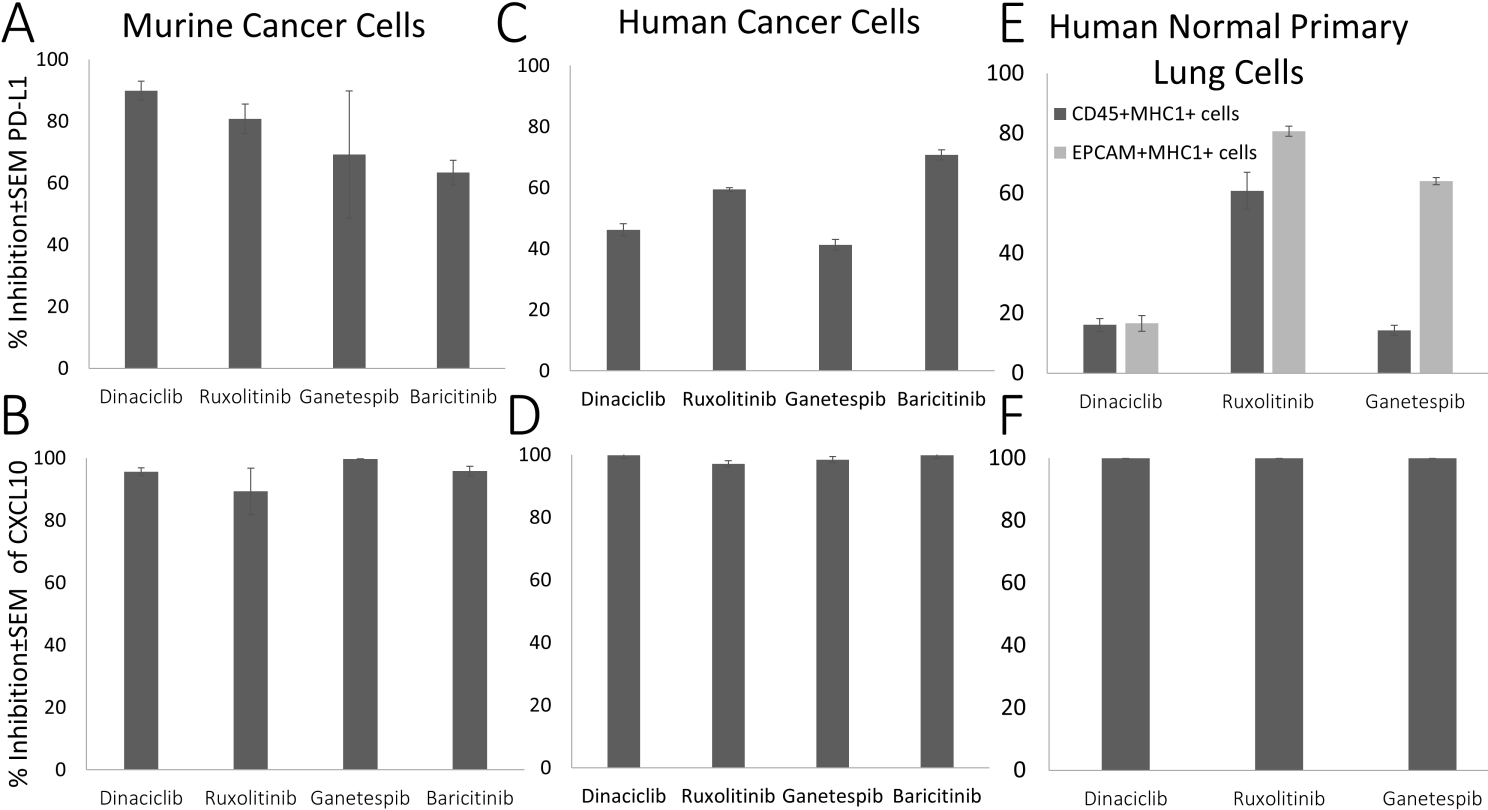
Suppression (% of inhibition) of IFNγ-dependent PD-L1 and CXCL10 expression by selected drugs compared to IFNγ-treated cells. **(A, B)** Murine cancer cell; **(C, D)** Penc1 human cancer cells; **(E, F)** Human normal primary lung cells (CD45+, EPCAM).

Out of the 11 tested drugs, we found that only the drugs of Group 1, Ruxolitinib, Baricitinib, Dinaciclib and Ganetespib, demonstrated significant inhibition of IFNγ-dependent PD-L1 (59.4 ± 0.62, 70.7 ± 1.63, 46.2 ± 1.93 and 41.29 ± 1.68% inhibition, respectively) and CXCL10 expression (97 ± 2.9, 99 ± 0.2, 100 ± 0.25 and 98 ± 0.62% inhibition, respectively) in Panc1 human pancreatic cancer cells (**Figure 7**) compared to cells treated only with IFNγ. This effect was previously observed in mice cancer cells.

We further tested the effect of the Group 1 drugs on human primary lung cells. Treatment with Ruxolitinib (a JAK/STAT pathway inhibitor which is known to have an effect on IFN-γ pathway), Dinaciclib and Ganetespib significantly inhibited IFNγ-dependent PD-L1 expression in human normal primary lung CD45+MHC1+ immune cells (72.5, 18.2 and 12.75% inhibition, respectively) and in human primary EPCAM-MHC1+ lung epithelial cells (81.3, 19.6 and 64.11% inhibition, respectively), and completely eliminated CXCL10 expression (100% inhibition for all drugs) compared to their expression levels in control IFNγ-treated cells.

### *In-vivo* assessment of hits in a DTH mouse model

The delayed-type hypersensitivity (DTH) model serves as a rodent model for studying inflammation mediated by soluble antigens, primarily involving the activation of CD4+ or CD8+ T cells. These reactions are characterized by the release of mediators from activated T cells, which subsequently stimulate local endothelial cells and recruit macrophages, resulting in localized inflammation and swelling.

To assess the impact of the hits on inflammation and immune cell recruitment *in-vivo*, we utilized the DTH mouse model in BALB/c mice. The mice were treated with ten selected compounds (out of the 11 tested on human cells). Our results showed that the only compounds significantly reducing ear swelling and immune cell infiltration *in-vivo*, were the three compounds that decreased both IFNγ-dependent PD-L1 and CXCL10 expression levels across all cell types tested *in-vitro* (because JAK/STAT pathway inhibitors are well known to have this effect on IFNγ pathway, we chose only one of the two JAK/STAT pathway inhibitors that we found in the screening as a positive control).

**Figure 8:**
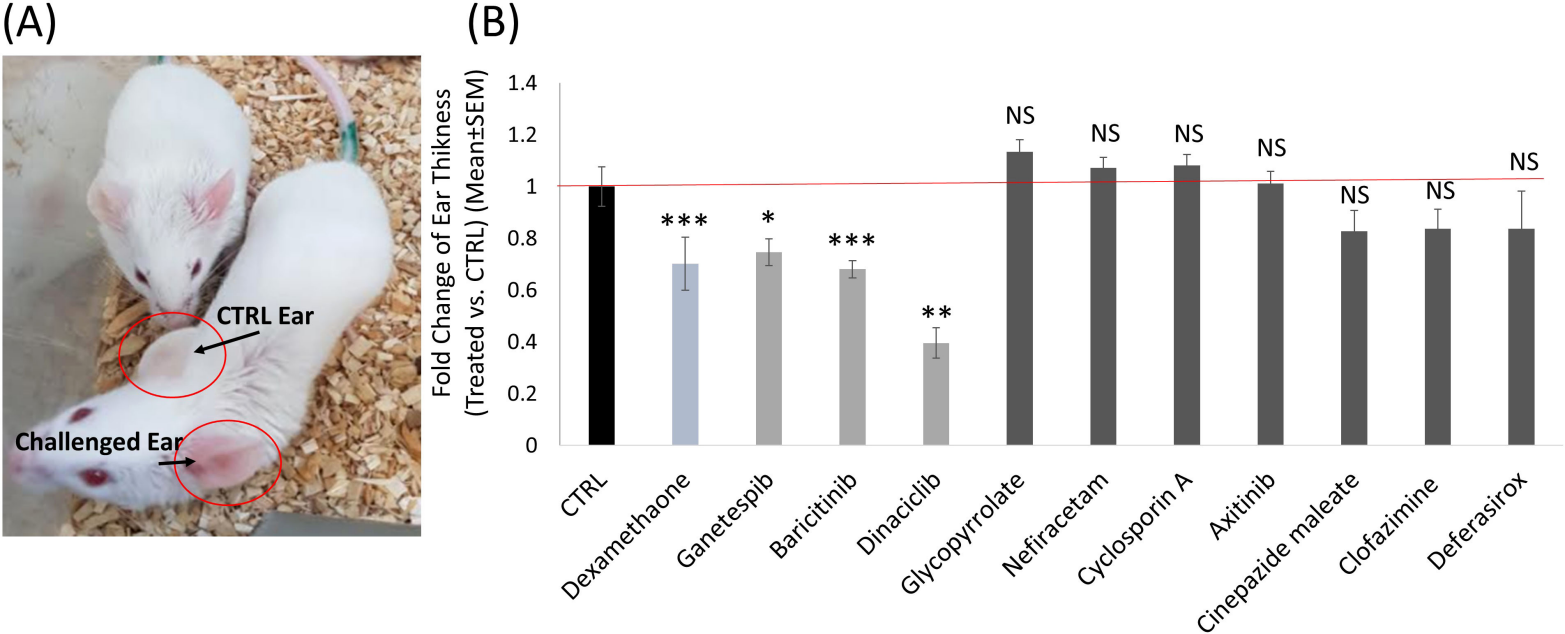
The effect of selected drug candidates on inflammation in a DTH mouse model. **(A)** Image depicting two control DTH mice. The challenged left ear exhibits redness and swelling compared to the control right ear. This image represents the DTH mouse model. **(B)** Histogram demonstrating the impact of the different selected candidate drugs, compared to the negative control group (Black) and the positive control group treated with Dexamethasone (Teal). Ganetespib, Baricitinib and Dinaciclib were the three candidate drugs that significantly (P<0.01) decreased ear swelling in the DTH mouse model (Light Grey). ns, not significant statistically p>0.05; *, significant statistically p<0.05; **, significant statistically p<0.01; ***, significant statistically p<0.001.

Compared to the control mice, we found three candidate drugs, all from Group 1, that decrease ear swelling: Baricitinib, 32% decrease (± 3%, P<0.0001); Ganetespib, 25% decrease (± 5%, P=0.01); and Dinaciclib decreased swelling by 60% (± 6%, P<0.001) (**Figure 8**). These are comparable to the decrease in ear swelling by that Dexamethazone (± 10%, P=0.0001), used as positive control. Thus, we speculated that these compounds could serve as promising candidates for the treatment of inflammation, conditions characterized by cytokine storm, such as COVID-19, and autoimmune disorders.

Of the other seven compounds tested, none exhibited a significant effect on ear swellin.

## Discussion

Our study highlights the cell-dependent nature of IFNγ-mediated immune responses and demonstrates the feasibility of manipulating these responses through drug intervention using a phenotypic screening approach. We propose a novel screening platform capable of identifying new molecules or repurposing known drugs for the treatment of immune dysfunctions, including cancer, viral infections, autoimmune diseases, and graft-versus-host disease (GVHD). Through our investigation, we screened a library of 1496 drugs using this technology and identified 50 compounds that modulate the expression IFNγ-mediated genes. Further evaluation of three selected compounds *in-vitro* on human primary lung cells and *in-vivo* in a DTH mouse model revealed their potential as candidates for treating hyperinflammatory syndrome in viral infections such as COVID-19, inflammation and autoimmune disorders.

IFNγ is a type II interferon that triggers antiviral and adaptive immune responses through a JAK-STAT signaling pathway. IFNγ converts the STAT1 homodimers into an antiparallel configuration. The reoriented STAT1 homodimers translocate to the nucleus, where they bind to GAS sites on the primary response genes, including IRF1, which activates a large number of secondary response genes. These genes carry out a range of immunomodulatory functions (27).

COVID-19 severity may be imparted due to a dysregulated inflammatory response (28). Baricitinib, an orally administered, selective inhibitor of JAK 1 and 2, was predicted with the use of artificial intelligence (AI) algorithms to be a potential therapeutic against against severe acute respiratory syndrome coronavirus 2 (SARS-CoV-2) (29–31). Moreover, Stebbing et al. recently identified that Baricitinib exerts an antiviral and anti-cytokine effect in hospitalized patients with COVID-19 pneumonia and in spheroid models of SARS-CoV-2 infection (32).

Based to this, identifying a JAK/STAT pathway inhibitor such as Baricitinib in our screen, as an anti-inflammatory compound, was not surprising and strengthened the validity and reliability of our novel HTS setup. The results of the studies mentioned above along with our DTH results, suggest that both Dinaciclib and Ganetespib, which demonstrated a similar effect as Baricitinib, should be further evaluated as good candidates for treatment of hyperinflammatory syndrome in viral infections (such as in COVID-19), inflammation and autoimmune disorders.

Dinaciclib (also known as MK-7965 and SCH727965) is a cyclin-dependent kinase (CDK1/2/5/9) inhibitor that controls cell-cycle progression and induces apoptosis in different tumor cells. Dinaciclib inhibits phosphorylation in retinoblastoma and also inhibits *in-vitro* cell growth of pancreatic cancer cells. It has been shown to be clinically active in refractory chronic lymphocytic leukemia and serves as a good treatment for several tumors. Tumors that intrinsically lack antigen presentation or are devoid of T cells that can respond to antigens are significantly less likely to respond to anti-PD1 (17). Thus, therapies that can create an immunogenic environment within tumors that otherwise are immune-suppressed or immunologically barren have the potential to expand the number of patients who could benefit from anti-PD1 treatment (33). Enhancing the immunogenicity of the tumor with an immunogenic cell death (ICD) inducer such as Dinaciclib can augment the overall efficacy of anti-PD1 checkpoint blockade. Hossain et al. found that Dinaciclib induces ICD by stimulating, *In vitro* and *in vivo*, the early expression of type I IFN response genes, and enhances anti-PD1–mediated tumor suppression (33). In addition, it was found that CDK1/2/5/9 inhibition overcomes IFNγ-mediated adaptive immune resistance in pancreatic cancer (34).

In addition to its anti-tumor effect, it was found that Dinaciclib has a strong antiviral activity that was observed across two cell lines (Vero E6 and A549-ACE2) (35). CDK5 was also found as an inducer for glutamyl-prolyl tRNA synthetase phosphorylation and activation of the IFNγ-activated inhibitor of translation pathway and, thus, suppresses inflammatory gene expression by translational control (36). Takahashi et al. showed that inhibition or absence of CDK5 evokes anti-inflammatory effects and activation of macrophages (37). In our screening, we found that Dinaciclib inhibits the IFNγ-dependent expression of CXCL10 in a mouse pancreatic cell line as well as in human normal primary lung cells and decreases inflammation *in-vivo* in the DTH mouse model. These findings, along with other findings, such as those mentioned above, suggest a promising repurposing of Dinaciclib for other immune disorders such as hyperinflammatory syndrome in viral infections (such as in COVID-19), inflammation and autoimmune disorders.

According to the existing literature, within the immune system, CDK5 has been implicated in IFNγ-induced PD-L1 upregulation, which allows certain cells to evade detection by the immune system. Decreased CDK5 expression led to increased expression of the PD-L1 transcriptional repressors IRF2 and IRF2BP and consequent decreased PD-L1 expression (38, 39). These findings could suggest Dinaciclib’s mechanism of action.

In addition to Baricitinib and Dinaciclib, Ganetespib was also found to have anti-inflammatory characteristics both *in-vitro* and *in-vivo*. Ganetespib (STA-9090) is a heat shock protein 90 (Hsp90) inhibitor which exhibits potent cytotoxicity in a wide variety of hematological and solid tumor cell lines (40, 41). Ganetespib causes depletion of receptor tyrosine kinases, extinguishing of downstream signaling, inhibition of proliferation and induction of apoptosis (40). In addition, Ganetespib possesses superior JAK/STAT inhibitory activity to both P6 and 17-allylamino-17-demethoxygeldanamycin (17-AAG) in terms of potency or duration of response in the HEL92.1.7 cells (42, 43). HSP90 inhibitors were found to robustly decrease PD-L1 surface expression, through a mechanism that appears to involve the regulation of master transcriptional regulators (i.e., STAT-3 and c-Myc) which might explain its mechanism of action (44). It was also found that Hsp90 regulate PD-L1 expression via HER2/PI3K/AKT signaling pathway which might suggest another option for the mechanism of PD-L1 downregulation by HSP-90 inhibitor (45). Moreover, it was reported that Hsp90 inhibitors may possess a potential therapeutic utility for a number of inflammatory autoimmune diseases (MRL/LPR mouse model for systemic lupus erythematosus and models for rheumatoid arthritis) (46, 47). Hsp90 also plays key roles in some stages of the virus life cycle (48).

In addition, it was found that Ganetespib inhibits inflammatory cytokine production in ex-vivo stimulated lymphocytes. Recently, Lilja et al. found that Ganetespib suppresses lipopolysaccharide-induced (LPS) lung inflammation *in-vivo* and that it suppresses LPS-induced neutrophil mobilization into the blood, as well as neutrophil and mononuclear cell-rich steroid-refractory lung inflammation (49). Taken together with our study’s results, this suggests that Ganetespib may serve as an effective treatment not only for cancer but also for autoimmune disorders, inflammation and hyperinflammatory syndrome in viral infections, and should be further tested for the treatment of COVID-19 infection.

In this study we used a mouse pancreatic cell line and a mouse PD-L1 promotor for the screening assay in order to identify drugs that potentially can be used for human patients. This was necessary for further analysis of hits *in-vivo*. Yet, variability in signaling pathways and cell cycle dynamics may also affect drug sensitivity and resistance mechanisms in different cells and different species. Nevertheless, we further validated the hits’ potential in human cancer cell line, human primary lung epithelial and human primary lung immune cells. Several types of human cancer cell lines can be further analyzed using our platform.

Many studies focus on targeting tumor cells while underestimating the role of the tumor microenvironment, including immune evasion mechanisms and stromal interactions that contribute to therapy resistance. In this study we have focused on the IFN-γ response that has an effect on the tumor microenvironment and immune response.

This platform can be efficient in finding new drugs or repurposing FDA approved drugs for cancer therapy. Despite upregulation and indication across many cancers, the predicted response rate to anti-PD-1/PD-L1 therapy remains 20–30%. Even if patients did initially respond to therapy, many patients developed resistance and relapsed following treatment (50). Tumors that express IFN-γ, CXCL10, and PD-L1 have a better chance to respond well to anti PD-1/PD-L1 therapy and are considered immunogenic (20). Tumors or immune environment lacking PD-L1 expression often exhibit primary resistance to checkpoint inhibition, as the absence of PD-L1 expression suggests a lack of pre-existing T cell activation. Paradoxically, upregulating PD-L1 expression (associated with immune suppression) in tumors that do not express it, creates a targetable immune-suppressive mechanism, allowing anti-PD-L1 therapy to re-activate anti-tumor T cells. When PD-L1 is artificially upregulated, it indicates that T cells are now interacting with the tumor cells. This shifts the tumor microenvironment from “cold” to “hot” immunogenic state. Therefore, drugs that upregulated IFN-γ dependent CXCL10 and PD-L1 expression in our screening may be considered as good candidates for cancer immunotherapy. Researching drugs that upregulate IFNγ-dependent PD-L1 expression, offers a promising strategy in cancer therapy. This approach can improve anti-tumor immune responses and potentially overcome resistance mechanisms associated with low PD-L1 expression (51). This requires further validation in *in-vivo* tumor models.

In conclusion, the phenotypic screening approach facilitated by our novel screening platform has proven effective in immunomodulating drug discovery. Through this method, we have identified promising compounds capable of targeting IFNγ-dependent immune dysregulation, holding potential for the treatment of various immune-related disorders. Moving forward, our platform can be expanded to conduct HTS assays on larger libraries of compounds, thereby accelerating the identification of novel drug candidates with therapeutic implications for immune-related conditions.

## Data Availability

The datasets presented in this study can be found in online repositories. The names of the repository/repositories and accession number(s) can be found in the article/**Supplementary Material**.
