# Supplementary figures and images for: Identification of Dinaciclib and Ganetespib as anti-inflammatory drugs using a novel HTP screening assay that targets IFNγ-dependent PD-L1
Source: Front Immunol. 2025 Apr 8;16:1502094. doi: 10.3389/fimmu.2025.1502094 (PMC12011776; doi:10.3389/fimmu.2025.1502094)

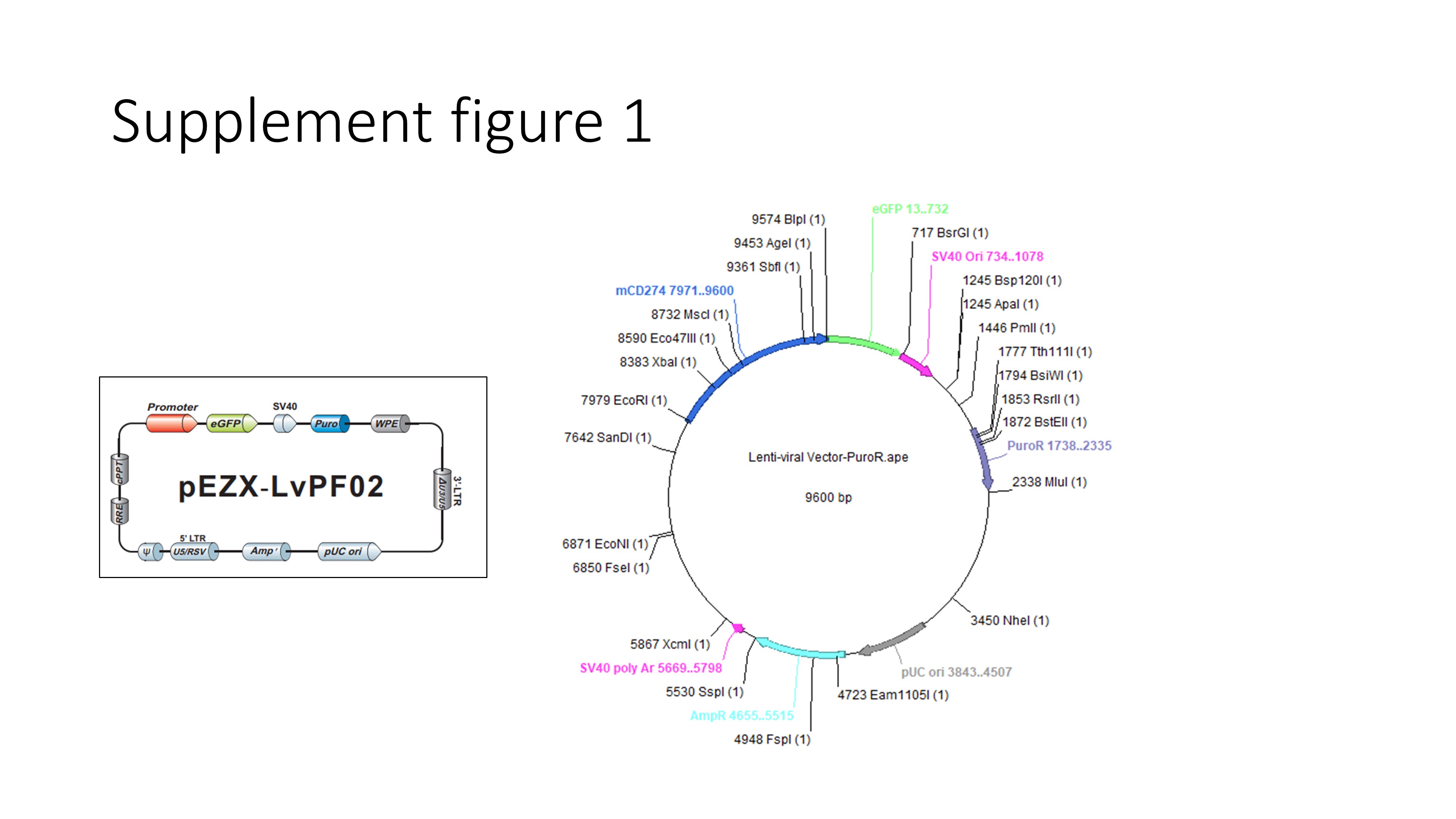

Supplement: Supplementary file 1 [file Image1.jpeg]
