# Supplementary material for: Identification of Dinaciclib and Ganetespib as anti-inflammatory drugs using a novel HTP screening assay that targets IFNγ-dependent PD-L1
Source: Front Immunol. 2025 Apr 8;16:1502094. doi: 10.3389/fimmu.2025.1502094 (PMC12011776; doi:10.3389/fimmu.2025.1502094)
Supplement: Supplementary file 2 [file DataSheet1.pdf]

Lenti-viral vector PD-L1 Promotor-eGFP

AGCCACCATGGTGAGCAAGGGCGAGGAGCTGTTCACCGGGGTGGTGCCCATCCTGGTCGA
GCTGGACGGCGACGTAAACGGCCACAAGTTCAGCGTGTCCGGCGAGGGCGAGGGCGATGC
CACCTACGGCAAGCTGACCCTGAAGTTCATCTGCACCACCGGCAAGCTGCCCGTGCCCTG
GCCCACCCTCGTGACCACCCTGACCTACGGCGTGCAGTGCTTCAGCCGCTACCCCGACCA
CATGAAGCAGCACGACTTCTTCAAGTCCGCCATGCCCGAAGGCTACGTCCAGGAGCGCAC
CATCTTCTTCAAGGACGACGGCAACTACAAGACCCGCGCCGAGGTGAAGTTCGAGGGCGA
CACCCTGGTGAACCGCATCGAGCTGAAGGGCATCGACTTCAAGGAGGACGGCAACATCCT
GGGGCACAAGCTGGAGTACAACTACAACAGCCACAACGTCTATATCATGGCCGACAAGCA
GAAGAACGGCATCAAGGTGAACTTCAAGATCCGCCACAACATCGAGGACGGCAGCGTGCA
GCTCGCCGACCACTACCAGCAGAACACCCCCATCGGCGACGGCCCCGTGCTGCTGCCCGA
CAACCACTACCTGAGCACCCAGTCCGCCCTGAGCAAAGACCCCAACGAGAAGCGCGATCA
CATGGTCCTGCTGGAGTTCGTGACCGCCGCCGGGATCACTCTCGGCATGGACGAGCTGTA
CAAGTAGCTCGAGCTGTGGAATGTGTGTCAGTTAGGGTGTGGAAAGTCCCCAGGCTCCCC
AGCAGGCAGAAGTATGCAAAGCATGCATCTCAATTAGTCAGCAACCAGGTGTGGAAAGTC
CCCAGGCTCCCCAGCAGGCAGAAGTATGCAAAGCATGCATCTCAATTAGTCAGCAACCAT
AGTCCCGCCCCTAACTCCGCCCATCCCGCCCCTAACTCCGCCCAGTTCCGCCCATTCTCC
GCCCCATGGCTGACTAATTTTTTTTATTTATGCAGAGGCCGAGGCCGCCTCTGCCTCTGA
GCTATTCCAGAAGTAGTGAGGAGGCTTTTTTGGAGGCCTAGGCTTTTGCAAAAAGCTCCC
GGGAGCTTGTATATCCATTTTCGGATCTGATCGGCGCGGGCCGCGATCCCGCCCCTCTCC
CTCCCCCCCCCCTAACGTTACTGGCCGAAGCCGCTTGGAATAAGGCCGGTGTGCGTTTGT
CTATATGTTATTTTCCACCATATTGCCGTCTTTTGGCAATGTGAGGGCCCGGAAACCTGG
CCCTGTCTTCTTGACGAGCATTCCTAGGGGTCTTTCCCCTCTCGCCAAAGGAATGCAAGG
TCTGTTGAATGTCGTGAAGGAAGCAGTTCCTCTGGAAGCTTCTTGAAGACAAACAACGTC
TGTAGCGACCCTTTGCAGGCAGCGGAACCCCCCACCTGGCGACAGGTGCCTCTGCGGCCA
AAAGCCACGTGTATAAGATACACCTGCAAAGGCGGCACAACCCCAGTGCCACGTTGTGAG
TTGGATAGTTGTGGAAAGAGTCAAATGGCTCTCCTCAAGCGTATTCAACAAGGGGCTGAA
GGATGCCCAGAAGGTACCCCATTGTATGGGATCTGATCTGGGGCCTCGGTGCACATGCTT
TACATGTGTTTAGTCGAGGTTAAAAAAACGTCTAGGCCCCCCGAACCACGGGGACGTGGT
TTTCCTTTGAAAAACACGATGATAAGCTTGCCACAACCCACAAGGAGACGACCTTCCATG
ACCGAGTACAAGCCCACGGTGCGCCTCGCCACCCGCGACGACGTCCCCCGGGCCGTACGC
ACCCTCGCCGCCGCGTTCGCCGACTACCCCGCCACGCGCCACACCGTCGACCCGGACCGC
CACATCGAGCGGGTCACCGAGCTGCAAGAACTCTTCCTCACGCGCGTCGGGCTCGACATC
GGCAAGGTGTGGGTCGCGGACGACGGCGCCGCGGTGGCGGTCTGGACCACGCCGGAGAGC

GTCGAAGCGGGGGCGGTGTTCGCCGAGATCGGCCCGCGCATGGCCGAGTTGAGCGGTTCC

CGGCTGGCCGCGCAGCAACAGATGGAAGGCCTCCTGGCGCCGCACCGGCCCAAGGAGCCC

GCGTGGTTCCTGGCCACCGTCGGCGTCTCGCCCGACCACCAGGGCAAGGGTCTGGGCAGC

GCCGTCGTGCTCCCCGGAGTGGAGGCGGCCGAGCGCGCCGGGGTGCCCGCCTTCCTGGAG

ACCTCCGCGCCCCGCAACCTCCCCTTCTACGAGCGGCTCGGCTTCACCGTCACCGCCGAC

GTCGAGGTGCCCGAAGGACCGCGCACCTGGTGCATGACCCGCAAGCCCGGTGCCTAGACG

CGTCTGGAACAATCAACCTCTGGATTACAAAATTTGTGAAAGATTGACTGGTATTCTTAA

CTATGTTGCTCCTTTTACGCTATGTGGATACGCTGCTTTAATGCCTTTGTATCATGCTAT

TGCTTCCCGTATGGCTTTCATTTTCTCCTCCTTGTATAAATCCTGGTTGCTGTCTCTTTA

TGAGGAGTTGTGGCCCGTTGTCAGGCAACGTGGCGTGGTGTGCACTGTGTTTGCTGACGC

AACCCCCACTGGTTGGGGCATTGCCACCACCTGTCAGCTCCTTTCCGGGACTTTCGCTTT

CCCCCTCCCTATTGCCACGGCGGAACTCATCGCCGCCTGCCTTGCCCGCTGCTGGACAGG

GGCTCGGCTGTTGGGCACTGACAATTCCGTGGTGTTGTCGGGGAAGCTGACGTCCTTTCC

ATGGCTGCTCGCCTGTGTTGCCACCTGGATTCTGCGCGGGACGTCCTTCTGCTACGTCCC

TTCGGCCCTCAATCCAGCGGACCTTCCTTCCCGCGGCCTGCTGCCGGCTCTGCGGCCTCT

TCCGCGTCTTCGCCTTCGCCCTCAGACGAGTCGGATCTCCCTTTGGGCCGCCTCCCCGCC

TGGAATTAATTCTGCAGTCGAGACCTAGAAAAACATGGAGCAATCACAAGTAGCAATACA

GCAGCTACCAATGCTGATTGTGCCTGGCTAGAAGCACAAGAGGAGGAGGAGGTGGGTTTT

TCCAGTCACACCTCAGGACCTTTAAGACCAATGACTTACAAGGCAGCTGTAGATCTTAGC

CACTTTTTAAAAGAAAAGAGGGGACTGGAAGGGCTAATTCACTCCCAACGAAGACAAGAT

CTGCTTTTTGCCTGTACTGGGTCTCTCTGGTTAGACCAGATCTGAGCCTGGGAGCTCTCT

GGCTAACTAGGGAACCCACTGCTTAAGCCTCAATAAAGCTTGCCTTGAGTGCTTCAAGTA

GTGTGTGCCCGTCTGTTGTGTGACTCTGGTAACTAGAGATCCCTCAGACCCTTTTAGTCA

GTGTGGAAAATCTCTAGCAGTAGTAGTTCATGTCATCTTATTATTCAGTATTTATAACTT

GCAAAGAAATGAATATCAGAGAGTGAGAGGCTAGCGTTTTACCGTCGACCTCTAGCTAGA

GCTTGGCGTAATCATGGTCATAGCTGTTTCCTGTGTGAAATTGTTATCCGCTCACAATTC

CACACAACATACGAGCCGGAAGCATAAAGTGTAAAGCCTGGGGTGCCTAATGAGTGAGCT

AACTCACATTAATTGCGTTGCGCTCACTGCCCGCTTTCCAGTCGGGAAACCTGTCGTGCC

AGCTGCATTAATGAATCGGCCAACGCGCGGGGAGAGGCGGTTTGCGTATTGGGCGCTCTT

CCGCTTCCTCGCTCACTGACTCGCTGCGCTCGGTCGTTCGGCTGCGGCGAGCGGTATCAG

CTCACTCAAAGGCGGTAATACGGTTATCCACAGAATCAGGGGATAACGCAGGAAAGAACA

TGTGAGCAAAAGGCCAGCAAAAGGCCAGGAACCGTAAAAAGGCCGCGTTGCTGGCGTTTT

TCCATAGGCTCCGCCCCCCTGACGAGCATCACAAAAATCGACGCTCAAGTCAGAGGTGGC

GAAACCCGACAGGACTATAAAGATACCAGGCGTTTCCCCCTGGAAGCTCCCTCGTGCGCT

CTCCTGTTCCGACCCTGCCGCTTACCGGATACCTGTCCGCCTTTCTCCCTTCGGGAAGCG

TGGCGCTTTCTCATAGCTCACGCTGTAGGTATCTCAGTTCGGTGTAGGTCGTTCGCTCCA
AGCTGGGCTGTGTGCACGAACCCCCCGTTCAGCCCGACCGCTGCGCCTTATCCGGTAACT
ATCGTCTTGAGTCCAACCCGGTAAGACACGACTTATCGCCACTGGCAGCAGCCACTGGTA
ACAGGATTAGCAGAGCGAGGTATGTAGGCGGTGCTACAGAGTTCTTGAAGTGGTGGCCTA
ACTACGGCTACACTAGAAGAACAGTATTTGGTATCTGCGCTCTGCTGAAGCCAGTTACCT
TCGGAAAAAGAGTTGGTAGCTCTTGATCCGGCAAACAAACCACCGCTGGTAGCGGTTTTT
TTGTTTGCAAGCAGCAGATTACGCGCAGAAAAAAAGGATCTCAAGAAGATCCTTTGATCT
TTTCTACGGGGTCTGACGCTCAGTGGAACGAAAACTCACGTTAAGGGATTTTGGTCATGA
GATTATCAAAAAGGATCTTCACCTAGATCCTTTTAAATTAAAAATGAAGTTTTAAATCAA
TCTAAAGTATATATGAGTAAACTTGGTCTGACAGTTACCAATGCTTAATCAGTGAGGCAC
CTATCTCAGCGATCTGTCTATTTCGTTCATCCATAGTTGCCTGACTCCCCGTCGTGTAGA
TAACTACGATACGGGAGGGCTTACCATCTGGCCCCAGTGCTGCAATGATACCGCGAGACC
CACGCTCACCGGCTCCAGATTTATCAGCAATAAACCAGCCAGCCGGAAGGGCCGAGCGCA
GAAGTGGTCCTGCAACTTTATCCGCCTCCATCCAGTCTATTAATTGTTGCCGGGAAGCTA
GAGTAAGTAGTTCGCCAGTTAATAGTTTGCGCAACGTTGTTGCCATTGCTACAGGCATCG
TGGTGTCACGCTCGTCGTTTGGTATGGCTTCATTCAGCTCCGGTTCCCAACGATCAAGGC
GAGTTACATGATCCCCCATGTTGTGCAAAAAAGCGGTTAGCTCCTTCGGTCCTCCGATCG
TTGTCAGAAGTAAGTTGGCCGCAGTGTTATCACTCATGGTTATGGCAGCACTGCATAATT
CTCTTACTGTCATGCCATCCGTAAGATGCTTTTCTGTGACTGGTGAGTACTCAACCAAGT
CATTCTGAGAATAGTGTATGCGGCGACCGAGTTGCTCTTGCCCGGCGTCAATACGGGATA
ATACCGCGCCACATAGCAGAACTTTAAAAGTGCTCATCATTGGAAAACGTTCTTCGGGGC
GAAAACTCTCAAGGATCTTACCGCTGTTGAGATCCAGTTCGATGTAACCCACTCGTGCAC
CCAACTGATCTTCAGCATCTTTTACTTTCACCAGCGTTTCTGGGTGAGCAAAAACAGGAA
GGCAAAATGCCGCAAAAAAGGGAATAAGGGCGACACGGAAATGTTGAATACTCATACTCT
TCCTTTTTCAATATTATTGAAGCATTTATCAGGGTTATTGTCTCATGAGCGGATACATAT
TTGAATGTATTTAGAAAAATAAACAAATAGGGGTTCCGCGCACATTTCCCCGAAAAGTGC
CACCTGACGTCGACGGATCGGGAGATCAACTTGTTTATTGCAGCTTATAATGGTTACAAA
TAAAGCAATAGCATCACAAATTTCACAAATAAAGCATTTTTTTCACTGCATTCTAGTTGT
GGTTTGTCCAAACTCATCAATGTATCTTATCATGTCTGGATCAACTGGATAACTCAAGCT
AACCAAAATCATCCCAAACTTCCCACCCCATACCCTATTACCACTGCCAATTACCCTGTG
GGCGCAATTAACCCTCACTAAAGGGAACAAAAGCTGGAGCTGCAAGCTTAATGTAGTCTT
ATGCAATACTCTTGTAGTCTTGCAACATGGTAACGATGAGTTAGCAACATGCCTTACAAG
GAGAGAAAAAGCACCGTGCATGCCGATTGGTGGAAGTAAGGTGGTACGATCGTGCCTTAT
TAGGAAGGCAACAGACGGGTCTGACATGGATTGGACGAACCACTGAATTGCCGCATTGCA
GAGATATTGTATTTAAGTGCCTAGCTCGATACATAAACGGGTCTCTCTGGTTAGACCAGA

TCTGAGCCTGGGAGCTCTCTGGCTAACTAGGGAACCCACTGCTTAAGCCTCAATAAAGCT

TGCCTTGAGTGCTTCAAGTAGTGTGTGCCCGTCTGTTGTGTGACTCTGGTAACTAGAGAT

CCCTCAGACCCTTTTAGTCAGTGTGGAAAATCTCTAGCAGTGGCGCCCGAACAGGGACTT

GAAAGCGAAAGGGAAACCAGAGGAGCTCTCTCGACGCAGGACTCGGCTTGCTGAAGCGCG

CACGGCAAGAGGCGAGGGGCGGCGACTGGTGAGTACGCCAAAAATTTTGACTAGCGGAGG

CTAGAAGGAGAGAGATGGGTGCGAGAGCGTCAGTATTAAGCGGGGGAGAATTAGATCGCG

ATGGGAAAAAATTCGGTTAAGGCCAGGGGGAAAGAAAAAATATAAATTAAAACATATAGT

ATGGGCAAGCAGGGAGCTAGAACGATTCGCAGTTAATCCTGGCCTGTTAGAAACATCAGA

AGGCTGTAGACAAATACTGGGACAGCTACAACCATCCCTTCAGACAGGATCAGAAGAACT

TAGATCATTATATAATACAGTAGCAACCCTCTATTGTGTGCATCAAAGGATAGAGATAAA

AGACACCAAGGAAGCTTTAGACAAGATAGAGGAAGAGCAAAACAAAAGTAAGACCACCGC

ACAGCAAGCGGCCGGCCGCTGATCTTCAGACCTGGAGGAGGAGATATGAGGGACAATTAA

TTGGAGAAGTGAATTATATAAATATAAAGTAGTAAAAATTGAACCATTAGGAGTAGCACC

CACCAAGGCAAAGAGAAGAGTGGTGCAGAGAGAAAAAAGAGCAGTGGGAATAGGAGCTTT

GTTCCTTGGGTTCTTGGGAGCAGCAGGAAGCACTATGGGCGCAGCGTCAATGACGCTGAC

GGTACAGGCCAGACAATTATTGTCTGGTATAGTGCAGCAGCAGAACAATTTGCTGAGGGC

TATTGAGGCGCAACAGCATCTGTTGCAACTCACAGTCTGGGGCATCAAGCAGCTCCAGGC

AAGAATCCTGGCTGTGGAAAGATACCTAAAGGATCAACAGCTCCTGGGGATTTGGGGTTG

CTCTGGAAAACTCATTTGCACCACTGCTGTGCCTTGGAATGCTAGTTGGAGTAATAAATC

TCTGGAACAGATTTGGAATCACACGACCTGGATGGAGTGGGACAGAGAAATTAACAATTA

CACAAGCTTAATACACTCCTTAATTGAAGAATCGCAAAACCAGCAAGAAAAGAATGAACA

AGAATTATTGGAATTAGATAAATGGGCAAGTTTGTGGAATTGGTTTAACATAACAAATTG

GCTGTGGTATATAAAATTATTCATAATGATAGTAGGAGGCTTGGTAGGTTTAAGAATAGT

TTTTGCTGTACTTTCTATAGTGAATAGAGTTAGGCAGGGATATTCACCATTATCGTTTCA

GACCCACCTCCCAACCCCGAGGGGACCCGACAGGCCCGAAGGAATAGAAGAAGAAGGTGG

AGAGAGAGACAGAGACAGATCCATTCGATTAGTGAACGGATCTCGACGGTATCGCCTTTA

AAAGAAAAGGGGGGATTGGGGGGTACAGTGCAGGGGAAAGAATAGTAGACATAATAGCAA

CAGACATACAAACTAAAGAACTACAAAAACAAATTACAAAAATTCAAAATTTTCGGGTTT

ATTACAGGGACAGCAGAGATCCAGTTTATCTAATACGACTCACTATAGGGAGAGAGAGAG

AATTACCCTCACTAAAGGGAGGAGAAGCATGAATTGAAGGAGATAGAACCAGATCTTGGA

ATTCACTAAGCCAGCGGACACCCCAGTATTCACCCAGTGCACTACTTTGGAATAGTAGTT

TTAGTAAGTAAGTGGGGGAAAGCAGAGAATGAAGAAGGCCCTTGAAGTCCAACAGTGAAA

TGTTTAAAGATGACAGTGCTCTGTGGAGTTCCCAAGGTTTTGTCTTGGAAAAAGTCCACA

CTTCCAGTTCGCAGAAAGTCTTTCTCAACATCATTTAGAATAGACTTCCCCCACCTGGAT

CCCGAGACTGGCCGTGATCCACAGCGTTCACAAAGGGCACGGTTCGAGATGGGAAGTTCT

TGAACGGCAAGACAACTGGTTTCATTATGTCGAGGAACTTTGAGGAAGTCACCAAATCCA

CGATTTAAAAATATATTTCCTATTATACAGACACACCTACTTTCTAGAATTAAAACTGAG

TCATTTGCTTGATATTAACTCTATAGGTTGTATAACTCTATATGTAAAGTCATGTCAAGA

CTGTCACGTATCCACGTATCCAGAAAGGGCTTGAAAGAGATGGGGAATCGGATGGTAATT

TGAAGTGTCTGGATTCTGAAGATAAAATTTAAGTCAGAGATCTTATGACTTCAGATATTT

TGCTTCTAAAGCGCTCACTGCTCAAGCCTGAAGATTTGAAATTCGGGTCCTCATTACCCA

TAATAAATGCAGTGATGGCCCATTTCTGAGACCCTAGCCCTGGCAGCAGGGGCGCGGATG

GGGATCCCTGGACCACGCTGGCCGGCTAGTTTGGCCAGCTGCGAGCCCGAGGTTAGGTAA

GAGAGACCCTCTTTCAAAAATCAAGGTGGGAGCTGTAGAGGAAGGCAACCTATGTGGATC

TCCAAGCACACGCTCCCCCCCACCCCCACCCCCGACCTCAGGTTCCACTCCCACCCAAAA

TAGAGCTGAGTTGTTTACTCTGGACTGTTTCTTTGAGGGAACCTGATTTACAAGAAAGCT

AATGCAGGTTTCACTTTCACTTTTAGTTTCGTTTTTAAATAGTGTTTGTTTGTTTTTGTT

TTTATCGACAGCCTCTCAGTAGCAGCCCGGTTGTCTTGGAGCTCTCTCTATAGACCAGAG

ACTCACCTGCCACTGGCTCCTGAGTACTGGAATTAAGGCGTGTGTCACCGCACCGAAGCC

TAGTTTCGTTTTTTCTTAAACTGTGAATATCCCAAAGCTGACTCTAAAGTCATCCGCAGG

AAATACTATGAGATAAACTCATGCTCAAAGGGACTGGGTGGCTTCGGTTTCACAGACAGC

GGAGGTTGGACAAGGCTTCCGCGGAGTGGGCGGGGCTCTGAACTCGAGATAAGACCAGGA

AATCGTGGTCCCCAAGCCTCATGCCAGGCTGCACTTGCACGTCGCGGGCCAGTCTCCTCG

CCTGCAGGTAAGGGAGCATCTTCTCGCGGAATCCGCTTGCAGGGCACTTTAAAGAGCCAG

AATCCCTAGACCTTTTTAGGACGGAGAAGGGAACCGGTTTCCTGGGAAAGTTAAGAACTC

AGAATCCGCAGTTTTGTGTGTTTATGGATCTTGTGGGTAGGTAGCTGGGTCAGAAGAGAT

GAATTAATTGGTCCTAGCGCGACTTGACTGTTTGCTAAGCTTGGTACCGAGCTCGGATCC
